# GPR120, a potential therapeutic target for experimental colitis in IL-10 deficient mice

**DOI:** 10.18632/oncotarget.14210

**Published:** 2016-12-26

**Authors:** Jie Zhao, Honggang Wang, Peiliang Shi, Wenbo Wang, Ye Sun

**Affiliations:** ^1^ Department of General Surgery, The First Affiliated Hospital of Soochow University, Suzhou, 215000, Jiangsu, China; ^2^ Department of General Surgery, Taizhou People's Hospital, Medical School of Nantong University, Taizhou, 225300, Jiangsu, China; ^3^ MOE Key Laboratory of Model Animal for Disease Study, Model Animal Research Center, Nanjing University, Nanjing, 210000, Jiangsu, China; ^4^ Department of Orthopedics, The First Affiliated Hospital of Soochow University, Suzhou, 215000, Jiangsu, China; ^5^ Orthopedic Institute, Soochow University, Suzhou, 215000, China

**Keywords:** Crohn's disease, colitis, docosahexaenoic acid, GPR120, TAK1/IKK-α/IkB-α/p65 pathway

## Abstract

It has been proved that interleukin-10-knockout (IL-10 KO) mice display the most similar characteristics to that of human Crohn's disease (CD). Docosahexaenoic acid (DHA) has well established beneficial effects on human and animal models health with potent anti-inflammatory effects with poorly understood mechanisms. This study was aimed at figuring out whether DHA could ameliorate the Crohn's colitis by activating GPR120 and whether GPR120 could be a potential therapeutic target for CD.16 week-old mice included in our present study were divided into three groups, WT group, IL-10 KO group and DHA group(IL-10 KO mice with DHA treatment, i.g., 35.5mg/kg/d), containing 8 mice in each group. The severity of colitis, pro-inflammatory cytokines concentrations, the expression/distribution of protein GPR120 and TAK1/IKK-α/IkB-α/p65 pathway in the proximal colons were evaluated at the end of the experiment. Administration of DHA showed promising results in the experimental chronic colitis (demonstrated by reduced infiltration of inflammatory cells, lowered inflammation scores, decreased pro-inflammatory cytokines) and body weight loss improvement. Moreover, in the DHA-treated mice, enhanced expression and improved distribution integrity of protein GPR120 were observed, which was probably associated with the regulation of TAK1/IKK-α/IkB-α/p65 pathway. Our results indicated that triggering GPR120 via the inhibition of TAK1/IKK-α/IkB-α/p65 pathway might be an important target for Crohn's colitis.

## INTRODUCTION

Crohn disease (CD) is a chronic inflammatory bowel disease (IBD) involves the entire gastrointestinal tract with no certain etiology [1]. It has been proved that interleukin-10-knockout (IL-10 KO) mice (generated by gene targeting) display the most similar characteristics to that of human CD [2]. The IL-10 KO mice mostly suffer from anemia, growth retardation and chronic colitis under specific pathogen-free (SPF) conditions [3]. Previous data has revealed the close correlation between nutrition and CD. Exclusive Enteral Nutrition (EEN) therapy has been suggested as a dietary intervention for inducing CD remission by mucosal healing, gut microbiota and immune function modulation [4, 5].

Omega-3 fatty acids (w-3 FAs), mainly docosahexaenoic acid (DHA) and ecosapentaenoic acid (EPA), have well established beneficial effects on human and animal models health with potent anti-inflammatory effects [6]. The anti-inflammatory potential of w-3 FAs has been well investigated and have therapeutic potential in numerous diseases [7], including rheumatoid arthritis [8], periodontitis [9], cancer [10] and IBD [7], but the mechanisms involved are poorly understood. Many studies have been carried out to investigate the effect of w-3 PUFAs in human CD and no consensus has been made [11]. The anti-inflammatory actions have been widely certified in animal colitis models and vitro studies through various mechanisms [12]. Many candidate targets of w-3 FAs and their metabolites contribute to the explanation of their pro-resolving and anti-inflammatory effects, though the combination of these targets is also possible to explain their effects [13]. Nuclear receptor PPARγ and the G protein-coupled receptors (GPCRs) have been suggested to be the molecular targets for the anti-inflammatory effects of w-3 FAs reported by recent pharmacological studies [13, 14]. GPR120 is a member of the rhodopsin-like family of GPCRs and is proved to be highly conserved across many species [15]. GPR120 has attracted a lot of attention recently because it can be activated by w-3 FAs [16] and it has been detected in macrophages, dendritic cells, adipocytes, clara cells in bronchiole epithelium, and enteroendocrine L cells in colon [17]. Studies have shown that GPR120 is a functional w-3 FA receptor/sensor and it can mediate potent anti-inflammatory effects *in vivo* by repressing macrophage-induced tissue inflammation [18]. However, the role of GPR120 played in the chronic experimental colitis and the related signaling pathway still remain unknown. This study was aimed at figuring out whether DHA could ameliorate the Crohn's colitis by activating GPR120 and whether GPR120 could be a potential therapeutic target for CD by mediating the inflammatory pathway.

## RESULTS

### DHA administration improved the experimental chronic colitis and body weight loss in IL-10 deficient mice

**Figure 1:**
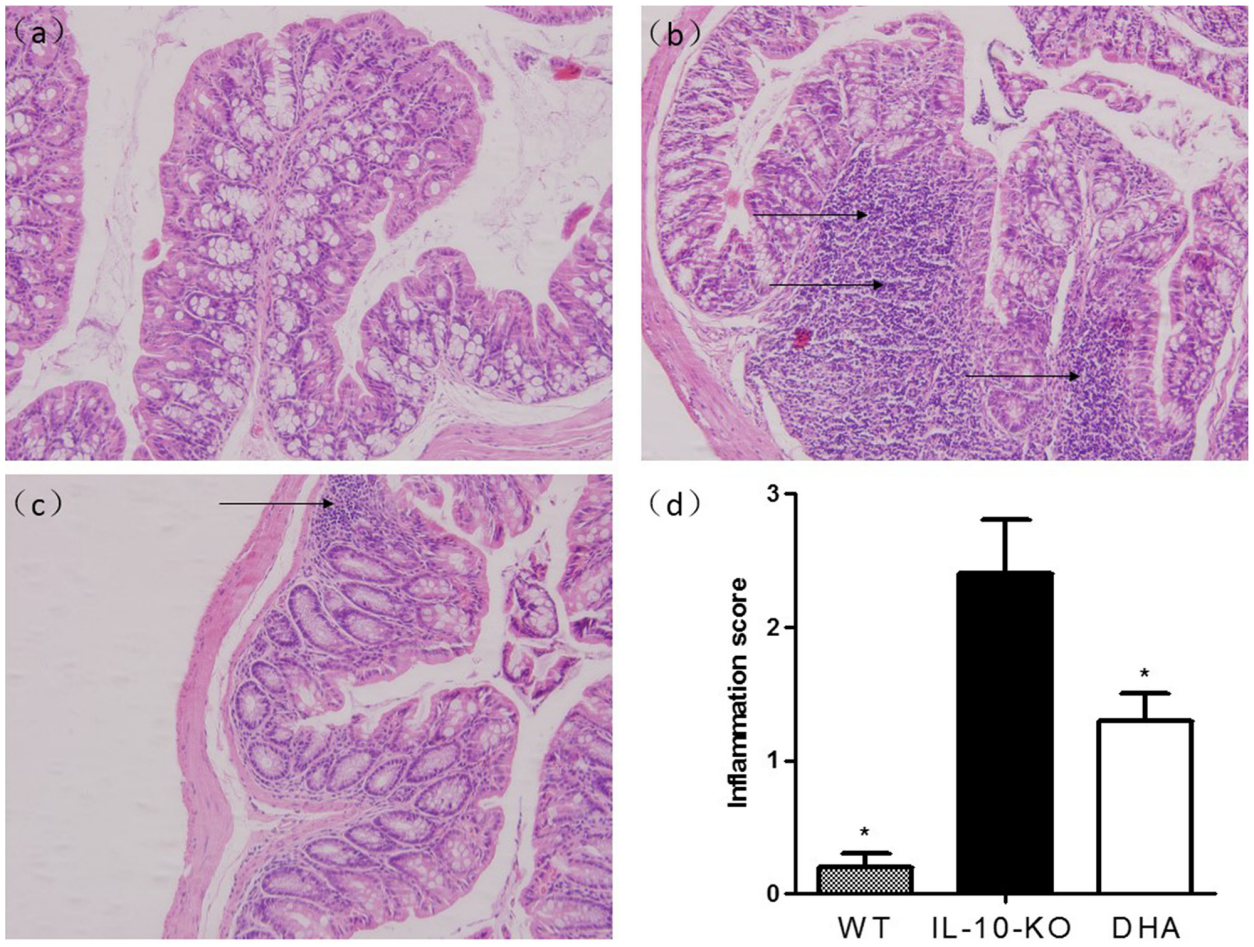
Changes in histological characterization and inflammation after DHA treatment in IL-10 knockout (KO) mice Histological sections of proximal colons in mice of three groups at the end of the experiment were presented, **a.** Colon of WT mouse, **b.** IL-10 KO mice with placebo treatment and **c.** IL-10 KO mice with DHA treatment. The results showed that DHA-treated mice showed markedly decreased inflammatory cells infiltration and much lower mean inflammation scores **d.** compared with placebo-treated IL-10 KO mice. Mean values were significantly different from those of the IL-10 KO group: *P<0.05. (n=8 per group).

**Figure 2:**
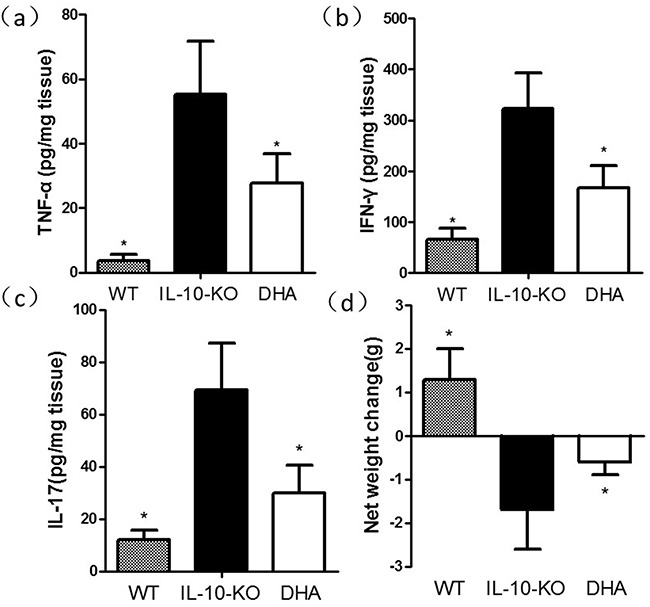
Therapeutic effect of DHA on the level of net weight change and colonic pro-inflammatory cytokines in IL-10 knockout (KO) mice by Enzyme-linked immunosorbent assay (ELISA) analysis Data are presented as means ± standard error of the mean (s.e.m.) (n=8 per group, *P<0.05 versus the IL-10 KO group mice).

All the included mice in our groups were survived. The tissue expressions of pro-inflammatory cytokines and histological features of colon were used for the evaluation of colitis severity. We firstly examined the histological changes of colon tissues in three groups. As expected, IL-10 KO mice showed more inflammatory cell infiltrations in the colonic mucosa in comparison with WT mice. In contrast, the infiltrate of lymphocytes was significantly improved and the histological appearance of the mucosa or submucosa was restored after DHA administration (see Figure 1). When compared with the placebo-treated IL-10 KO mice, the colonic inflammation scores in DHA group were significantly improved. In addition, DHA treatment resulted in reduced colonic inflammation as established decreased tissue concentrations of TNF-α, IFN-γ and IL-17, which was exhibited in Figure 2a-c. Additionally, data from Figure 2d indicated that the body weight loss was also observed in IL-10 KO mice and it was attenuated by DHA treatment.

### The effect of DHA therapy on GPR120

**Figure 3:**
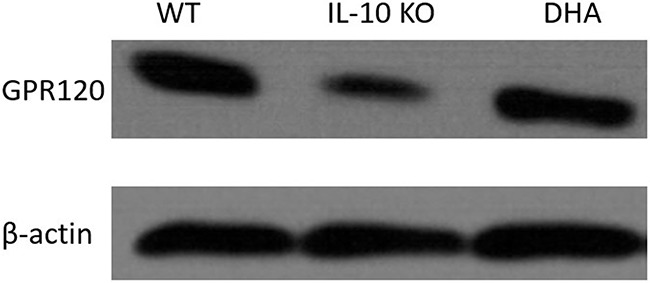
Western blot analysis of protein GPR120 expressions in proximal colon of mice in three groups The expressions of GPR120 were statistically analyzed relative to β-actin expression by densitometry.

**Figure 4:**
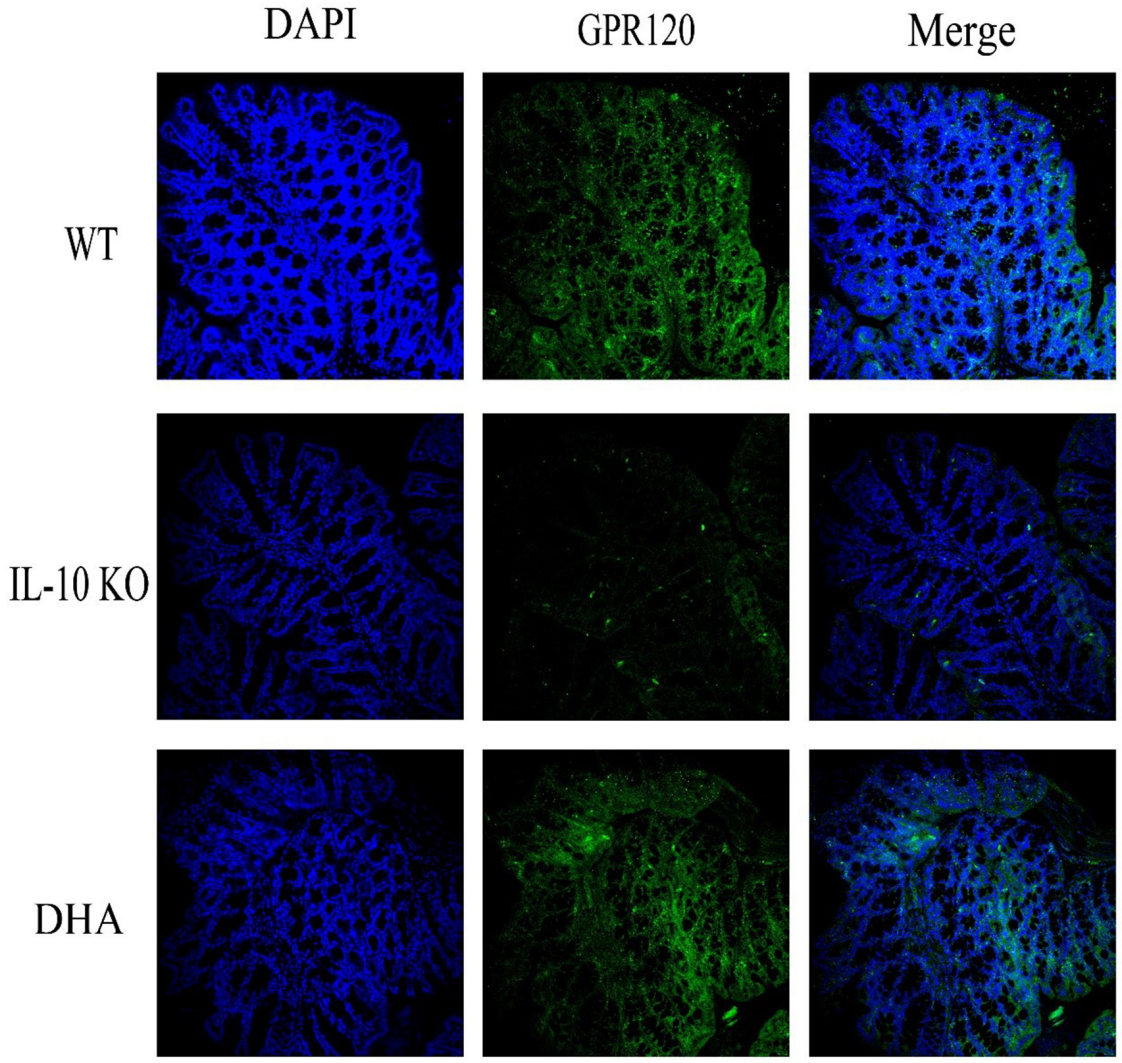
The expression and distribution of GPR120 in colon tissues Representative immunofluorescence (green) images of GPR120 and nuclei (blue) of proximal colon tissues in three groups (200× magnification). DHA treatment significantly improved the expressions and distribution integrity of GPR120 in proximal colon tissues.

To further investigate the impact of DHA therapy on GPR120 expressions in colon tissues, we subsequently performed Western blotting analysis. As shown in Figure 3, the expression of protein GPR120 in colonic mucosa of IL-10 KO mice was significantly decreased when compared with WT mice. Interestingly, the GPR120 expression in proximal colon tissues of IL-10 KO mice was significantly up-regulated by DHA treatment. Furthermore, the impaired continuity of the distribution and depressed expression of GPR120 in colon were also observed in placebo-treated IL-10 KO mice comparing with WT mice. However, DHA intervention partly reversed this change, as shown by immunofluorescence analysis in Figure 4.

### DHA repressed TAK1/IKK-α/IkB-α/p65 pathway

**Figure 5:**
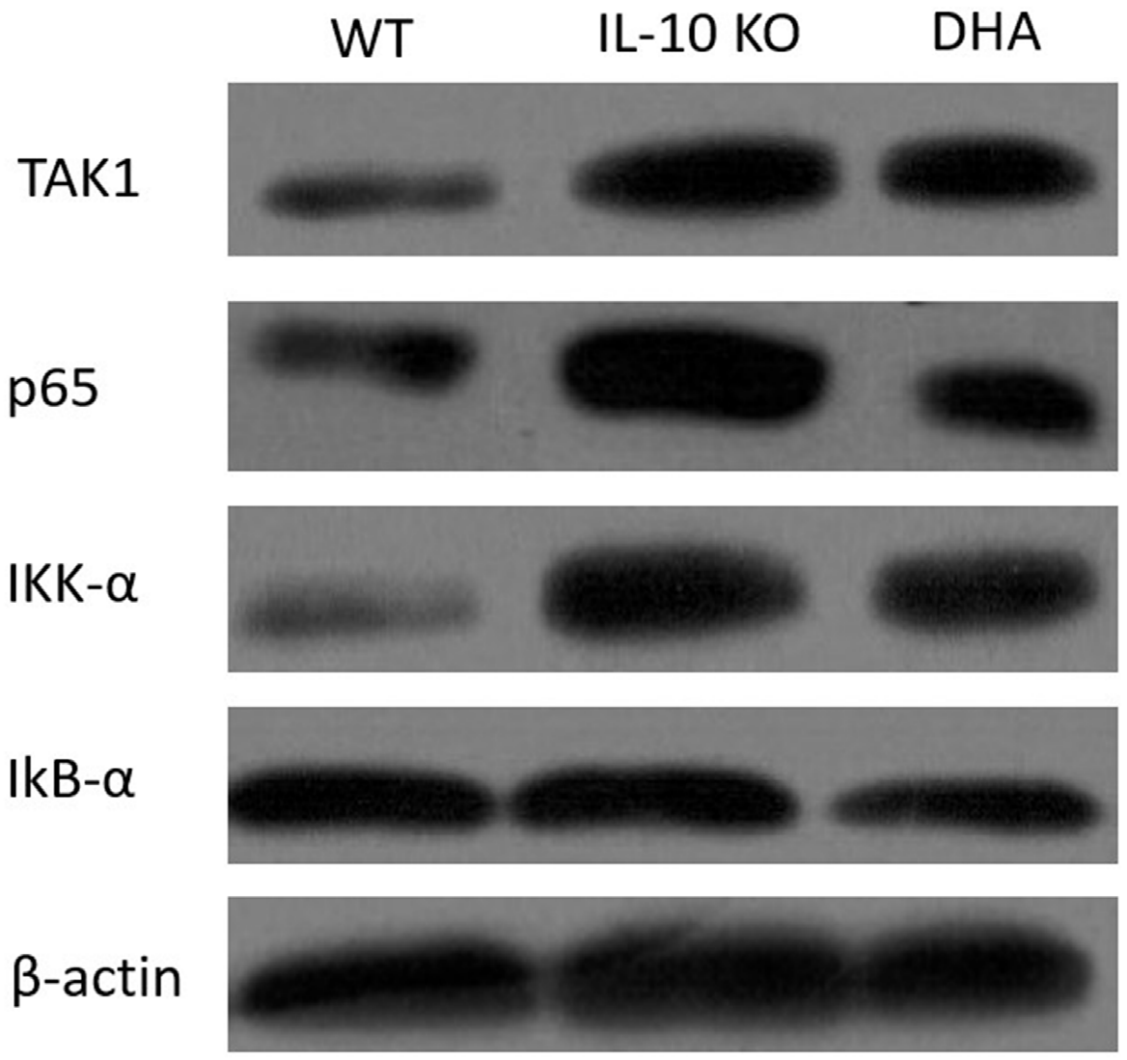
Western blot analysis of TAK1/IKK-α/IkB-α/p65 pathway associated proteins expressions in proximal colon of mice in three groups The expressions of TAK1, IKK-α, IkB-α and p65 were statistically analyzed relative to β-actin expression by densitometry.

**Figure 6:**
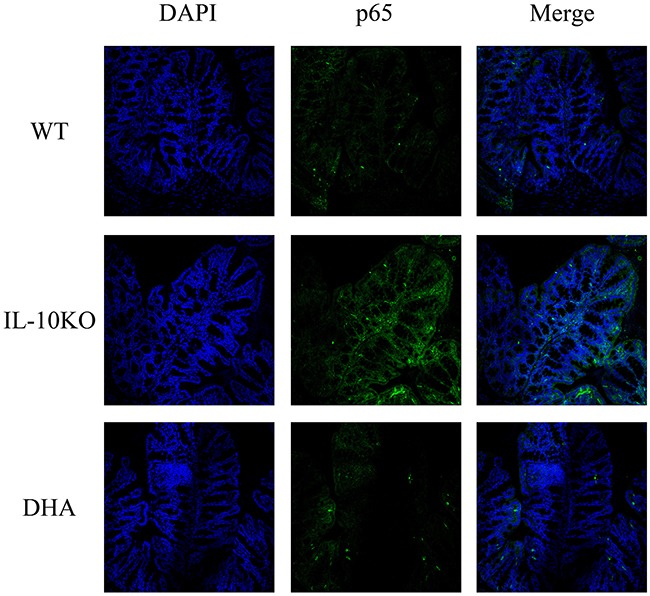
The expression and distribution of p65 in colon tissues Representative immunofluorescence (green) images of p65 and nuclei (blue) of proximal colon tissues in three groups (200× magnification). DHA treatment significantly decreased the expressions of p65 in proximal colon tissues.

At last, we evaluated TAK1/IKK-α/IkB-α/p65 pathway, which was closely associated with function of GPR120, in the proximal colons of mice in three groups. Our data indicated that the expressions of TAK1, IKK-α, IkB-α and p65 were significantly up-regulated in the IL-10 KO mice compared with the WT mice. In addition, a marked decrease in expressions of these proteins was observed in the colonic tissues from DHA-treated mice (see Figure 5). DHA treatment also significantly decreased the expression of p65 in proximal colon tissues, which was illustrated by immunofluorescence analysis in Figure 6.

## DISCUSSION

Previous studies in experimental models of inflammatory disease have already shown that w-3 PUFAs including DHA, EPA and α-linolenic acid (ALA) can serve as anti-inflammatory actions [11]. EPA and DHA can suppress inflammation by inhibiting the endotoxin-induced activation of nuclear factor-κB (NF-kB) in human monocytes [19]. It has been recently discovered that DHA can modulate the inflammatory response by decreasing cytokine production, dampening inflammation and actively promoting the resolution of inflammation [20]. DHA helps treat IBD in experimental models by inhibiting the nuclear factor-κB pathway with no certain mechanisms [11]. It has been reported that DHA exerts its anti-inflammatory effects through GPR120 agonism [18]. Studies conducted in bone marrow-derived mesenchymal stem cells show that EPA can also attenuate apoptosis by inducing adaptive autophagy via GPR120 [21]. Furthermore, ALA can also mediate phosphorylation of the human GPR120 receptor [14].

GPR120 is a bona fide long-chain fatty acid sensor of the rhodopsin family and its agonists is suggested to hold promise as a therapeutic target for diabetes, metabolic disorders and inflammatory diseases [22]. Mice deficient in GPR120 are more susceptible to heightened insulin resistance and higher expression of genes connected to inflammation [23]. In addition, the reduction of endogenous GPR120 protein levels leads to the abrogation of the anti-inflammatory effects of DHA, identifying GPR120 as one of the key mediators of the acute anti-inflammatory effects of DHA [24]. The evidence that GPR120 is a Gαq/ll GPR protein and can activate the PI3K/AKT and the PKC/MAPK/ERK cascades has been demonstrated by previous studies, however, these signaling components are likely dispensable for its anti-inflammatory actions [17]. Studies carried out in macrophages and dendritic cells suggest that GPR120 activation by DHA can interact with TAB1 via β-arrestin-2, and that this interaction interrupts TAK1 activation by LPS or TNF-α, suppressing inflammatory responses via NF-kB and JNK [18]. But whether GPR120 activation and its downstream mediators are involved in the anti-inflammatory effect of DHA in the experimental colitis has not been reported previously. In this present study, we identified the omega-3 FA receptor GPR120 as an anti-inflammatory mediator in the chronic colitis model of IL-10 KO mice.

Results from this study showed that triggering of GPR120 by DHA treatment ameliorate the experimental colitis in IL-10 deficient mice. The histopathological changes, reduction in inflammation score shown in Figure 1 and the decrease in pro-inflammatory cytokine expressions revealed in Figure 2 demonstrated that DHA obviously reversed the colitis in IL-10 KO mice. W-3 PUFAs can also significantly ameliorate the inflammation in chemically induced colitis models, including dextran sodium sulphate (DSS) [25], trinitro-benzene-sulfonic acid (TNBS) [26], and acetic acid-induced colitis [27]. To investigate whether the downstream mediators of GPR120 were involved in the anti-inflammatory effects of DHA in IL-10 KO mice, the expressions of TAK1, IKK-α, IkB-α and p65 were evaluated by western blotting. Results show that the expressions of proteins mentioned above were suppressed in DHA group compared with IL-10 KO mice, which indicated that the TAK1/IKK-α/IkB-α/p65 pathway was suppressed by DHA treatment. The expression of GPR120 evaluated by western blotting was up-regulated and the distribution continuity by immunofluorescence analysis was also significantly improved by DHA treatment. Taking the correlation between TAB1 and TAK1 into consideration, the stimulation of GPR120 receptor by DHA might recruit b-arrestin 2 to the cytosolic putative binding sites on GPR120, promote internalization of β-arrestin-2, which then formed a complex with TAB1, and hence the association of TAB1 with TAK1 was blocked. TNF-α couldn't activate TAK1 in the absence of TAB1, thereby blocking the downstream signaling to the IKK-α systems and hence inhibiting the downstream TNF-α inflammatory pathways [22]. Therefore, the block of inflammation pathway by DHA treatment was associated with the inhibition of TAK1 by GPR120 activation, which has been put forward by previous studies [23, 28].

Despite the GPR120-TAB1 interaction, GPR120 has also been reported to scaffold to NLRP3 (nucleotide-binding domain and leucine-rich repeat containing protein) through β2-arrestin to suppress the formation of the NLRP3 inflammasome by the activation of w-3 FA [29]. The activation of the NLRP3 inflammasome is generally pathogen-based and likely represents a GPR120-dependent mechanism more critical in anti-inflammatory effects of DHA. However, β2-arrestin is proved to have the capacity to scaffold hundreds of different proteins to the parental GPR in the recent discovery, it is likely that other GPR120 interactions mediating the anti-inflammatory effects of DHA will emerge with further investigation [30].

In summary, our study demonstrates that GPR120 significantly controls inflammation and raises the possibility that CD states. DHA decreases inflammation partially through inhibition of the NF-kB signaling pathway by activating GPR120. It adds a possible new mechanism to the existing mechanisms of DHA inhibition of inflammation and offers an effective therapeutic target for CD.

## MATERIALS AND METHODS

### Animals

16 week-old wild-type (WT) mice and IL-10 KO mice on a C57BL/6 background were purchased from the Jackson Laboratory (Bar Harbor, Maine). Mice were bred and maintained in a SPF (specific pathogen free) condition at the Model Animal Research Center of Nanjing University (Nanjing, China). All animal studies were carried out in accordance with the recommendations in the Guide for the Care and Use of Laboratory Animals of Nanjing University (Nanjing, China).

### Drug administration protocol

Mice included in our present study were divided into three groups, WT group (WT mice administered placebo), IL-10 KO group (IL-10 KO mice administered placebo) and treatment group (DHA group, IL-10 KO mice administered DHA), containing 8 mice in each group. IL-10 KO mice in the treatment group received DHA [31] (i.g., 35.5mg/kg/d, Cayman Chemical) treatment for two weeks, and the same volume of placebo was administrated to mice in WT and IL-10 KO groups. Mice were weighed every week and the dosages of DHA and placebo were adjusted according to the changed weight. The therapeutic effects of DHA were evaluated after the final drug administration. The weight of mice in each group before and after the treatment was recorded for the evaluation of net weight change.

### Histology

The histological procedure was performed to determine the inflammation status as described previously [32]. Proximal colons were obtained immediately and fixed in 10% buffer neutral formalin and embedded in paraffin after mice were euthanized. Then 6 um-thick sections were stained with haematoxylin and eosin (H&E). Two independent pathologists blinded to the study design gave the samples inflammation score containing the number of lesions and the severity of the disease. Each proximal colon segment was scored from 0 to 4 on the following well establish criteria described by Singh et al [33]. The summation of scores per mouse provided a total colonic disease score.

### Enzyme-linked immunosorbent assay (ELISA)

Protein extracts were obtained by homogenization of colonic segments in homogenization buffer consisting of a protease inhibitor to determinate the inflammatory cytokines in colonic mucosa. And the procedure was according to the description of the manufacturer's protocol in details. Cytokines including TNF-α, IL-1β and IFN-γ were measured by ELISA using DuoSet ELISA development kits (R&D systems, Minneapolis, MN).

### Immunofluorescence

Immunostaining was performed to determine the expressions of GPR120 and p65 as described previously ^(33)^. 6 um-thick frozen sections of proximal colon were transferred to coated slides, fixed in 1% paraformaldehyde, and washed 3 times with PBS. Thereafter, nonspecific binding was blocked with 5% normal goat serum in PBS. After incubation with monoclonal antibodies against GPR120 and p65 (Abcam, UK) in PBS with 1% goat serum overnight at 4 °C, sections were washed and incubated with Alexa 488-conjugated secondary antibodies for 60 min. Images were visualized using a confocal microscopy (OLYPUS).

### Western blotting

Western blotting of protein expressions was performed in details as described previously [34, 35]. The primary antibodies against TAK1, IKK-α, IkB-α and p65 were all purchased from Abcam. Relative changes in protein expression were estimated from the pixel density using UN-SCAN-IT version 6.1 normalized to β-actin, and the results were calculated as target protein expression/β-actin expression ratios.

### Statistical analysis

SPSS version 19.0 software (SPSS, Inc.) was used to perform the statistical analyses. The data were expressed as means with their standard errors (SEM). Single-factor variance ANOVA analyses were used to evaluate changes in groups. Results were considered statistically significant if *P* values were < 0.05.
